# Supplementary material for: Content-rich biological network constructed by mining PubMed abstracts
Source: BMC Bioinformatics. 2004 Oct 8;5:147. doi: 10.1186/1471-2105-5-147 (PMC528731; doi:10.1186/1471-2105-5-147)
Supplement: Additional File 5 — The original Chilibot query results of the term "long-term potentiation (LTP)" and 22 other terms, limiting the latest references analyzed to the years 1990, 1995, 2000, and 2004. [file 1471-2105-5-147-S5.bz2 › chilibotAdditionalFile5/ltp2004/html/TAU_CAMKII.html]

 


 **TAU** and **CAMKII** 
  
Found 20 abstracts in PubMed,  **20 abstracts were retrieved and analyzed**.  


---

 Search Google  |
 PDF files only 
|  EDU domain only 

---

**Interactive relationship** (e.g. stimulation, inhibition, etc)

- Calcium calmodulin dependent protein kinase II  [ **CAMKII** ]  phosphorylates  **tau**  at Ser 262 but only partially inhibits its binding to microtubules.  Ref: 8674537 FEBS Lett, 1996
- Calcium calmodulin dependent protein kinase II  [ **CAMKII** ]  CaM kinase II has been shown to phosphorylate  **tau**  in vitro in such a way to decrease its electrophoretic mobility.  Ref: 1312209 Brain Res Mol Brain Res, 1992
- Treatment of the brain slices with KN 62, an inhibitor of the autophosphorylation of  **CaMKII**  at Thr 286 287, produced approximately 60% inhibition in  **CaMKII**  activity and no significant effect on  **tau**  phosphorylation at Ser 262 356.  Ref: 11172803 FEBS Lett, 2001
- **tau**  self association was also significantly decreased by prior phosphorylation with calcium calmodulin dependent protein kinase II  [ **CAMKII** ] .  Ref: 7861153 J Neurochem, 1995
- These studies suggest i that in mammalian forebrain the cytosolic  **CaMKII**  activity is regulated mainly by PP 2A, ii that  **CaMKII**  is the major  **tau**  Ser 262 356 kinase in brain, and iii that a decrease in PP 2A PP 1 activities in the brain leads to hyperphosphorylation of  **tau**  not only by inhibition of its dephosphorylation but also by promoting the  **CaMKII**  activity.  Ref: 11172803 FEBS Lett, 2001
- Inhibition of PP 2A upregulates  **CaMKII**  in rat forebrain and induces hyperphosphorylation of  **tau**  at Ser 262 356.  Ref: 11172803 FEBS Lett, 2001
- To our knowledge, this is the first data showing in vivo that the activation of  **CaMKII**  induces both Alzheimer like  **Tau**  phosphorylation and behavioral disturbances.  Ref: 12123558 Chin Med J (Engl), 2002
- At 3 6 h, kinase assays specific for PKA and  **CaMKII**  showed no increased activity toward either  **tau**  or selective substrates.  Ref: 11121021 Proc Natl Acad Sci U S A, 2000
- We found that a specific  **CaMKII**  inhibitor, KN 93 20 micromol L... KN 92, accelerated the inactivation of I to  **tau**  fast 66.9 4.4 versus 43.0 4.4 ms, n=35.  Ref: 10532949 Circ Res, 1999
- Furthermore, in rat brain slices kept metabolically active in oxygenated artificial CSF, phosphorylation of  **tau**  at Ser 262 was i increased up to 120% in the presence of bradykinin, a  **CaMKII**  activator, and ii inhibited by approximately 35% in the presence of KN 62.  Ref: 9801171 FEBS Lett, 1998
- The calcium induced increases in  **tau**  phosphorylation are not dependent upon protein synthesis, nor are protein kinase C or calcium calmodulin dependent protein kinase II  [ **CAMKII** ]  involved in the response.  Ref: 10409701 J Biol Chem, 1999
- Both cAMP PK and  **CaMKII**  readily phosphorylated  **tau**  and slowed the migration of  **tau**  on sodium dodecyl sulfate containing polyacrylamide gels.  Ref: 1331323 J Neurochem, 1992
- Thus,  **CaMKII**  is a major  **tau**  Ser 262 kinase in mammalian brain.  Ref: 9801171 FEBS Lett, 1998
- These results suggested that  **CaMKII**  is involved in the phosphorylation of  **tau**  in Alzheimer s disease brain.  Ref: 14665412 Neurosci Lett, 2003
- Protein kinase C and calcium calmodulin dependent protein kinase II  [ **CAMKII** ]  phosphorylate three repeat and four repeat  **tau**  isoforms at different rates.  Ref: 9062903 Mol Cell Biochem, 1997
- Ser 262 in human recombinant  **tau**  protein is a markedly more favorable site for phosphorylation by  **CaMKII**  than PKA or PhK.  Ref: 9801171 FEBS Lett, 1998
- In vitro, both PP 2A and PP 1 dephosphorylated  **tau**  at Ser 262 356 that was phosphorylated with purified  **CaMKII** .  Ref: 11172803 FEBS Lett, 2001
- Recombinant human  **tau**  was phosphorylated at Thr212, Ser214, Ser262, and Ser356 by  **CaMKII** .  Ref: 14665412 Neurosci Lett, 2003
- Two dimensional phosphopeptide maps and sequential phosphorylation experiments indicate that cAMP PK phosphorylates sites in the human  **tau**  isoforms that are phosphorylated by  **CaMKII** , as well as unique sites that are not phosphorylated by  **CaMKII** .  Ref: 8384512 Brain Res, 1993
- Both cAMP dependent protein kinase cAMP PK and calcium calmodulin dependent protein kinase II  **CaMKII**  readily phosphorylated the three human  **tau**  isoforms, although cAMP PK phosphorylated them to a significantly greater extent than  **CaMKII** .  Ref: 8384512 Brain Res, 1993
- **Tau**  protein is phosphorylated by cyclic AMP dependent protein kinase and calcium calmodulin dependent protein kinase II  [ **CAMKII** ]  within its microtubule binding domains at Ser 262 and Ser 356.  Ref: 8687413 Biochem J, 1996
- Two dimensional phosphopeptide maps indicate that cAMP PK phosphorylates a site or sites on  **tau**  that are phosphorylated by  **CaMKII** , as well as a unique site or sites that are not phosphorylated by  **CaMKII** .  Ref: 1331323 J Neurochem, 1992
- However, cAMP PK phosphorylated  **tau**  to a significantly greater extent than  **CaMKII**  1.5 and 0.9 mol of 32P mol of  **tau** , respectively, and phosphorylation of  **tau**  by cAMP PK resulted in a greater shift to a more acidic, less heterogeneous pattern on two dimensional nonequilibrium pH gradient gels compared with  **CaMKII**  phosphorylation.  Ref: 1331323 J Neurochem, 1992

**Parallel relationship** (e.g. studied together, co-existance, homology, etc.)

- The phosphorylation of microtubule  **tau**  by  **CaMKII**  was investigated using tandem mass spectrometry MS MS.  Ref: 14665412 Neurosci Lett, 2003
- The effects of cyclic AMP dependent protein kinase cAMP PK or calcium calmodulin dependent protein kinase II  **CaMKII**  phosphorylation on the binding of bovine  **tau**  to tubulin and calpain mediated degradation of  **tau**  were studied.  Ref: 1331323 J Neurochem, 1992
- In this study we compared the phosphorylation of  **tau**  at Ser 262 by  **CaMKII** , PhK and PKA in vitro as determined by radioimmunoblots developed by the monoclonal antibody 12E8 which recognizes P Ser 262 and P Ser 356.  Ref: 9801171 FEBS Lett, 1998
- In this study we have evaluated six non PDPKs cyclic AMP dependent A kinase, calcium phospholipid dependent C kinase, casein kinase 1 CK 1, casein kinase 2 CK 2, calcium calmodulin dependent protein kinase II  [ **CAMKII** ] , and calcium calmodulin dependent protein kinase from rat cerebellum for their abilities to induce Alzheimer like epitopes on  **tau** .  Ref: 7532213 J Neurochem, 1995
- In situ hybridization of calcium calmodulin dependent protein kinase II  [ **CAMKII** ]  and  **tau**  mRNAs.  Ref: 1312209 Brain Res Mol Brain Res, 1992
- Thr212 and Ser214 are in the consensus phosphorylation sequence of  **CaMKII**  RXXS T, and non fetal type phosphorylation sites of  **tau** .  Ref: 14665412 Neurosci Lett, 2003
- The regulation of the activity of  **CaMKII**  by PP 1 and PP 2A, as well as the role of this protein kinase in the phosphorylation of  **tau**  protein in forebrain were investigated.  Ref: 11172803 FEBS Lett, 2001
- In addition, phosphorylation with cAMP PK or  **CaMKII**  of recombinant  **tau**  in which Ser 262, Ser 356 or both had been mutated to alanines, clearly demonstrated that cAMP PK and  **CaMKII**  were able to phosphorylate both sites.  Ref: 8687413 Biochem J, 1996
- **CaMKII**  phosphorylation of  **tau**  did not alter either of these parameters.  Ref: 1331323 J Neurochem, 1992
- The inhibition of the two phosphatase activities or PP 2A alone resulted in an approximately 2 fold increase in  **CaMKII**  activity and an approximately 8 fold increase in the phosphorylation of  **tau**  at Ser 262 356 in 60 min.  Ref: 11172803 FEBS Lett, 2001
- In this study, the ability of cyclic AMP dependent protein kinase cAMP PK and calcium calmodulin dependent protein kinase II  [ **CAMKII** ]   **CaMKII**  to phosphorylate  **tau**  at Ser 262, as well as Ser 356, is demonstrated by use of a monoclonal antibody 12E8 which has been shown to recognize  **tau**  when these sites are phosphorylated.  Ref: 8687413 Biochem J, 1996
